# Annotations

**Published:** 1894-08-25

**Authors:** 


### Annotations.

### Work for the Duke of York.

A few months ago the Duke of York succeeded the Duke of Edinburgh as Master of the Trinity House. By assuming this office Prince George became king of the lighthouse men who are under the jurisdiction of the Trinity House. Much sympathy has been evoked of late for miners, owing to the length of hours during which they have to labour, but a miner's lot must be paradise compared with that of the lighthouse men. A correspondent who has been inquiring into the subject, informs us that lighthouse men have to work seventeen and a half hours a day all the year round. No holidays are granted them without formal petition to the Trinity House, and such petitions are by no means always assented to. If granted the lighthouse men have to pay the railway fare of a substitute from London out of their own earnings. No one has the interest of sailors more at heart than Prince George, and there is no class more deserving of sympathy and consideration than lighthouse men and pilots. We commend these facts to the earnest consideration of the Duke of York, and hope he will take an early opportunity of investigating the circumstances referred to, with a view to reform. At this season of the year very many people resort to the seaside for relaxation and pleasure. The more intelligent of them might usefully enquire into the condition and circumstance, of the lighthouse men wherever they might find themselves, and devote a little of their leisure to bringing the grievances of this class to the notice of the public through the press. The circumstances of railway servants have been immensely ameliorated of late, but a railway accident cannot occur without all the facts being fully investigated. Lighthouse men and those they are charged with the duty of protecting, are less fortunately circumstanced from the fact, that it is often difficult, if not impossible, to investigate all the causes which have led to the loss of a particular ship. Twelve hours a day without intermission is bad enough, but seventeen and a half hours on duty out of twenty-four must make existence a misery, and to live in any proper meaning of that term, an impossibility. We hope the king of the lighthouse men will speedily free them from this system of slavery, which ought long ago to have been rendered impossible.

### The Dangerous Bicycle.

Not long ago a sensational paragraph appeared in the columns of the Press recounting the death of a lady from over exertion due to bicycling. The fatality was used as a warning to all ladies against the dangers of this means of locomotion for their sex. The medical papers, with more common sense, refused to countenance so illogical a warning. Quite recently a male member of the population has fallen a victim to bicycling when in possession of a weak heart. The Press on this occasion can strike no more original note of warning than to point out the evils of over exertion, especially to the weakly. If we descant on the dangers of the bicycling, we would dwell on the dangers of the bicycling not for the rider but for the foot passenger. At this time of the year how many delightful regions can be explored at a moderate expenditure that would be impossible without the useful two wheels. Exercise, change and interest, all within the reach of the narrowest means to the possessor of a bicycle. Ordinary prudence will provide against the few possible mishaps, and cycling may be regarded as one of the safest modes of travelling. Whilst the delightful means of scouring the country becomes more and more resorted to by both sexes, the poor pedestrian is in a fair way of having his peace destroyed, and his life daily imperilled. This is no exaggeration. The alarming bell or horn which suddenly assails our ears may act as a warning, but is by no means a guide. The startled pedestrian's first impulse is to move somewhere, and as a rule he moves in the most unexpected direction. A skilled and conscientious rider manages to pass the most bewildered passenger without harm; the timid, inexperienced, or careless rider too frequently harms himself and the passer-by. A little sensational paragraphing would do no harm here. Sooner or later, and the sooner the better, some means must be devised of rendering bicycling less of the nuisance and danger it is fast becoming to the public who are not riders. It is not extermination, but regulation, which should be aimed at.

### The Holiday Season.

Not infrequently the anticipation proves to be the best part of a holiday. Few things are indeed pleasanter than the planning out of routes, discussions on agreeable possibilities, the new fields yet to be explored, and even the collections of addresses of eligible furnished houses or eminently respectable lodgings—for all these share in a certain holiday atmosphere. The unmarried seem to have rather the best of it on these occasions, because they are unencumbered by any save strictly personal responsibility. But before the long-talked-of date of departure arrives, many most delightful plans are annually abandoned, either because funds will not allow, or the usual leave has been curtailed through the caprice of an employer or the exigencies of business. Again, something occurs at the last moment to render it probable that the traveller may receive a sudden recall, in which case the chief aim of a holiday is immediately defeated. There is no rest for the body unless the mind is free from worry, and the hourly prospect of interruption is as wearing as the interruption itself. The post-bag, too, is not infrequently an enemy to the holiday-maker, and an excellent and frequent postal delivery is rather detrimental than otherwise at a health-resort, and the majority of people would get more profit from a holiday of ordinary dimensions if they courageously placed themselves out of reach of the daily budgets of news which often contain some discordant or unrestful element, and in any case tend to recall the usual life which it is best to put aside for awhile. The complete cessation of the daily occupation which the annual holiday affords is necessary to all brain-workers, and none should attempt to make a sacrifice of this time of leisure, which will inevitably be paid for in the long run. Health and spirits are certain to fail before the next recurrence of the holiday season. Many a breakdown has been traced by the physician to the patient's omission of his usual change, having probably, as he thought, a good and sufficient reason for staying at home. Shirking a holiday seldom answers, and the anticipation, realisation, and even the retrospection of a successful one are substantially advantageous to the worker who aspires to keep a sound mind in a healthy body.

